# The mediating role of professional self-concept in how teacher professional development influences rural physical education teachers' work engagement: a moderated mediation analysis

**DOI:** 10.3389/fpsyg.2026.1767900

**Published:** 2026-03-03

**Authors:** Jihong Yan, Xinyu Dai

**Affiliations:** 1 Physical Education College, Jimei University, Xiamen, Fujian, China; 2 Department of Physical Education, Xiamen University of Technology, Xiamen, Fujian, China

**Keywords:** multi-group SEM, physical education teachers, professional self-concept, teacher professional development, work engagement

## Abstract

This study investigates how physical education (PE) teachers' perceptions of professional development (TPD) quality influence their work engagement, with professional self-concept as a mediator, while examining the moderating roles of teaching experience, disciplinary background, and training frequency. Data from 733 rural Chinese PE teachers were analyzed using structural equation modeling (SEM). Results revealed that TPD perception positively predicted work engagement (β = 0.176, *p* < 0.01), with professional self-concept mediating 63.3% of this effect. Education majors exhibited stronger direct (β = 0.675 vs. 0.529) and mediated effects than non-education majors. Multi-group analysis revealed a distinct, non-linear pattern across career stages: early-career teachers (1–5 years) showed a complex effect characterized by a suppressing mediation through professional self-concept (β = −1.210), while mid-career teachers (6–15 years) showed non-significant paths. In contrast, veteran teachers (>15 years) demonstrated the highest training responsiveness (total effect = 0.538). Training frequency followed an inverted U-curve, peaking at 1–2 sessions/year before declining at ≥3 sessions. These findings underscore the critical need for differentiated and context-sensitive TPD programs to enhance PE teachers' engagement, with design imperatives that address the unique challenges and needs of specific career stages and disciplinary backgrounds.

## 1 Introduction

Teacher burnout is a prevalent issue that negatively impacts educational quality and student physical health, particularly in underdeveloped regions (Harris et al., 2025). In the Chinese context, this challenge is exacerbated within the rural education system, where structural disparities in resources, support, and student demographics create a uniquely demanding professional environment for teachers (Wang et al., 2025; Sun et al., 2024; Yan and Dai, 2025). Existing studies (Admiraal and Røberg, 2023; Parkes et al., 2015; Vem et al., 2020) have identified key determinants of work engagement, including governmental/societal support, teaching environment, and demographic factors (e.g., age, gender). For rural teachers in China, these factors often manifest as pronounced challenges: policy support may be embodied in specific but sometimes insufficient compensation schemes (Ma and Lei, 2025), the teaching environment is frequently characterized by resource constraints, and professional isolation can be intense (Sun et al., 2024). However, rural physical education (PE) teachers exhibit divergent perceptions of subject-specific professional development (TPD) programs, influenced not only by generic factors like training content and logistical challenges (Behrman et al., 2008) but also by the degree to which such TPD resonates with and enhances their core professional identity as PE educators (Li et al., 2023). Individual factors such as career stage and educational background further shape these perceptions.

While some teachers experience burnout and reduced work engagement, others demonstrate sustained self-efficacy (Putwain and Embse, 2018) and resilience. This divergence points to the potential mediating role of internal psychological resources. Specifically, a teacher's professional self-concept—a multifaceted construct closely related to professional identity and self-worth—may determine how external challenges are appraised and navigated (Li et al., 2023; Sun et al., 2024). This divergence is theorized to stem from differences in professional self-concept (Rubio-Valdivia et al., 2024)—a multifaceted construct encompassing teachers' perceptions of their professional competencies, attributes, and goals. Such self-perceptions become particularly salient when teachers' professional identity conflicts with low occupational self-worth (Farber, 1984).

Friedman and Farber (1992) highlights that teachers with robust professional self-concepts exhibit superior stress management, lower burnout, higher job satisfaction, and sustained teaching accomplishments. This protective function is arguably even more critical in rural settings, where external job resources are often limited, making intrinsic psychological resources like a positive professional self-concept a key asset for sustaining engagement (Sun et al., 2024). High-quality, and contextually relevant TPD programs serve dual roles: reflecting policy support and providing knowledge/skill upgrades. For PE teachers, effective TPD should not only update pedagogical skills but also affirm and develop their professional autonomy and subject-specific expertise, which are core components of a strong professional self-concept (Zhang and Qiu, 2024). Consequently, work engagement emerges from the interplay of policy (TPD quality) and individual (professional self-concept) factors (Mahmoudi-Gahrouei et al., 2016). This dynamic is critical in PE contexts, where teachers often face challenges to their work engagement due to factors like stagnant student fitness outcomes and the marginalization of their subject. These challenges are often amplified in rural schools (Wang et al., 2025), making the investigation of compensatory mechanisms, such as TPD and professional self-concept, particularly urgent. Strengthening professional self-concept may counteract this trend by enhancing job satisfaction and reducing burnout (Li et al., 2023), thereby creating a more positive pathway for improving instructional quality and student outcomes.

Despite these insights, no study has examined professional self-concept as a mediator between TPD perceptions and work engagement among rural PE teachers—a gap requiring urgent attention amid China's demographic shifts and their profound impact on rural education systems. This study aims to: (a) validate the TPD–work engagement link in this specific context, and (b) pioneer an investigation of professional self-concept's mediating role, while exploring the moderating effects of career stage, disciplinary background, and training frequency.

### 1.1 Theory and hypotheses

Teacher professional development (TPD) operates as a dynamic ecosystem where effectiveness depends not only on macro-level support networks (e.g., national policies, school culture; Zhu, 2014; Zhu et al., 2018), but crucially on teachers' perception of TPD quality. For physical education teachers—a group with particularly high demands for professional updating—the perceived quality dimensions of TPD (e.g., curriculum relevance, trainer expertise, organizational support) directly determine participation outcomes (Laxdal et al., 2020). Current research demonstrates that when teachers perceive TPD as effectively addressing their developmental needs, this positive evaluation translates into enhanced work engagement, manifested through emotional vitality, cognitive focus, and sustained commitment in teaching practice (Wang and Chen, 2025; Wang et al., 2024). This mechanism operates through two distinct pathways: on one hand, high-quality TPD directly improves work efficacy by delivering cutting-edge knowledge (e.g., advancements in sports medicine) and practical skills (e.g., digital teaching tools); on the other hand, it indirectly boosts engagement levels by shaping teachers' professional identity (Salajegheh et al., 2020).

Professional self-concept (i.e., teachers' core self-perception of their professional roles and competencies) serves as the critical mediator in this process. For rural PE teachers in China, this self-concept is fundamentally shaped by the interplay between their subject-specific expertise and the unique constraints of the rural educational context. Drawing on identity construction theory, teachers' perception of TPD quality influences this construct through: (1) Competence affirmation pathway: When training content closely aligns with practical needs, it reinforces teachers' self-identification as “capable educators” (Pennington and Richards, 2016); (2) Value internalization pathway: Policy interpretation and standard clarification during training facilitate the transformation of organizational expectations into intrinsic professional beliefs (Gao et al., 2025). In the rural PE setting, these pathways are particularly salient: competence affirmation often relates to adapting skills to resource-limited environments, while value internalization may involve reconciling the marginalized status of PE with a sense of personal mission. Empirical evidence confirms the behavioral consequences of enhanced professional self-concept—Van Lankveld et al. (2017) found that teachers with strong professional self-concepts not only demonstrate greater willingness for classroom innovation but also maintain significantly more persistent work engagement than control groups.

Notably, the formation of professional self-concept depends both on external interventions like TPD and individual traits. This trait-dependent effect is particularly pronounced among physical education teachers: their professional self-esteem (Motallebzadeh and Kazemi, 2018) determines sensitivity to training outcomes, pedagogical belief systems (Berger and Lê Van, 2019) moderate the conversion of policy awareness into professional commitment, while critical thinking skills (Sheybani and Miri, 2019) influence the depth of training content internalization. These factors collectively constitute an “individual-context interaction framework” that governs TPD's impact on work engagement.

### 1.2 Overall hypothetical model

Given the above theoretical foundations and existing research in the literature on the link between perception of TPD, professional self-concept, and work engagement, this study hypothesizes that the perception of TPD and work engagement are mediated by professional self-concept. Specifically, the degree of perception of TPD would promote improvements in Physical education teachers' professional self-concept, which in turn would contribute to their work engagement. Based on these, we hypothesize that:

H1: Teachers' perception of TPD quality positively predicts their work engagement.

H2: Teachers' perception of TPD quality has a positive effect on their professional self-concept.

H3: Teachers' professional self-concept positively influences their work engagement.

H4: Teachers' professional self-concept mediate the relationship between perception of TPD and teachers' work engagement.

### 1.3 The moderating role of teaching experience, pedagogical background, and training frequency

The study found that the sample distribution across the three dimensions—disciplinary background, teaching experience, and number of training sessions—was suitable for multi-group structural equation modeling (MG-SEM) analysis, with significant differences in the mediating effects among subdivided sample groups, revealing limitations in the current training system. Accordingly, the sample was divided into subgroups: by Pedagogical Background into education majors (*n* = 642) and non-education majors groups (*n* = 91); by training frequency [including formal learning, training, and online meetings (Tsiotakis and Jimoyiannis, 2016)] into one-time (*n* = 299), two-time (*n* = 313), and three-or-more-times groups (*n* = 121); and by teaching experience into less-than-1-year (*n* = 235), 1-to-5-year (*n* = 147), 6-to-15-year (*n* = 232), and more-than-15-year groups (*n* = 119). Multi-group SEM analysis was then conducted to examine the moderating effects of demographic variables on mediating pathways and assess training effectiveness differences across teacher populations.

**Figure 1:**
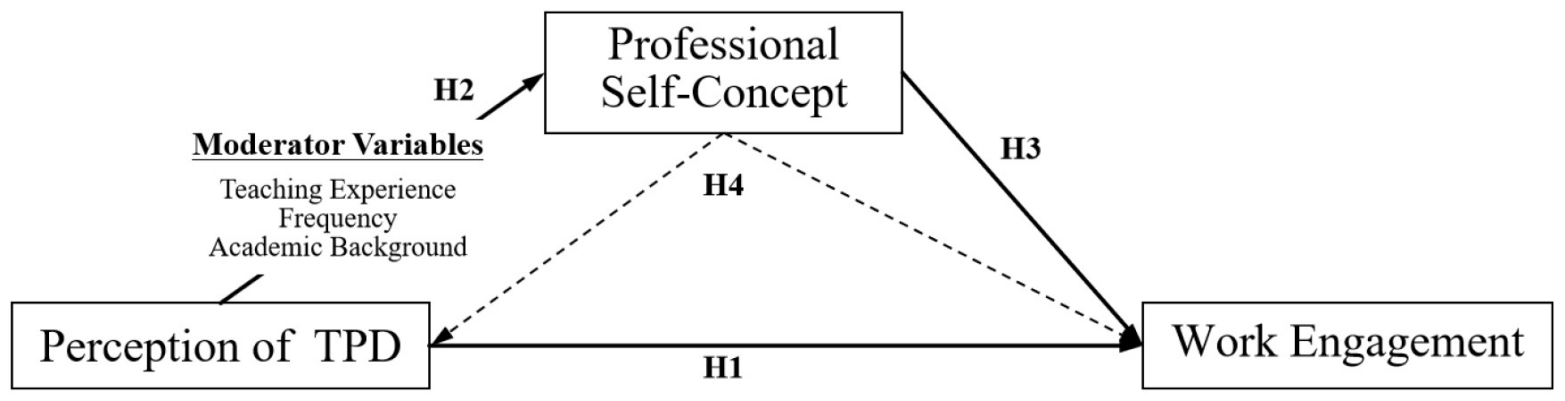
Research model.

Figure 1 shows a detailed hypothetical model of the mediating role of professional self-concept in the relationship between perception of TPD and work engagement.

## 2 Method

### 2.1 Procedure

This cross-sectional study employed a stratified random sampling method to ensure representativeness across different school levels. The primary stratification was based on school type (primary, junior high, high school). Within each stratum in Liaoning, Jilin, and Heilongjiang Provinces, schools were randomly selected with consideration for geographic distribution (prefecture-level cities vs. counties). Recruiting frontline teachers from 50 schools (25 primary, 18 junior high, and 7 high schools) across Liaoning, Jilin, and Heilongjiang Provinces in China. Before participation, the research team provided detailed explanations regarding the study's purpose, background, and expected duration. Questionnaires were administered only after obtaining informed consent, with a consent form attached to the first page. Participants who disagreed with the terms could select the “Disagree” option to exit the survey immediately.

Data collection was conducted through both field surveys and an online platform. To minimize common method bias, several procedural remedies were implemented during data collection: (a) ensuring respondent anonymity was emphasized in the instructions; (b) items for different constructs were presented in a counterbalanced order; and (c) scale items used varied anchor points and wording directions where appropriate. For the online component, survey hyperlinks were distributed to physical education directors at selected schools, who then forwarded them to eligible participants. From January to May 2025, a total of 850 questionnaires were distributed. After excluding invalid responses [e.g., those with excessive answer consistency or excessively short completion times (< 2 min)], 733 valid questionnaires were retained, yielding a response rate of 86.23%.

### 2.2 Sample

Data were collected from 850 teachers in 50 schools in three Chinese provinces. Our sample ultimately included 733 physical education teachers. The questionnaire included questions in four parts: demographic variables, perception of TPD, professional self-concept, and work engagement. On average, the participating teachers were 31.2 years old (*SD* = 9.20), had 9.60 years of experience (*SD* = 10.8), and 80.1% were male, which is related to the characteristics of the work of physical education teachers. Detailed data are shown in Table 1.

**Table 1:** Sample demographics.

**Variables**	**Category**	**Frequency**	**Percentage (%)**
Gender	Male	146	19.9
	Female	587	80.1
Teaching experience	≤ 1	235	32.1
	2–5	147	20.0
	6–15	232	31.6
	Above 15	119	16.3
Pedagogical background	Education majors	642	87.5
	Non-education majors	91	12.5
Frequency	1	299	40.7
	2	313	42.7
	≥3	121	16.6

*n* = 733.

### 2.3 Measures

#### 2.3.1 Professional self-concept

Teacher self-concept was measured using Friedman's scale (3), consisting of 23 items rated on a 6-point frequency scale (1 = never, 6 = always). Principal component analysis with varimax rotation revealed three factors, explaining 43% of the total variance. The scale demonstrated high internal reliability: Cronbach's α was 0.94 for the full scale and ranged from 0.77 to 0.91 for the subscales. In this study, the three factors of professional self-concept are interpreted and contextualized within the specific professional ecosystem of rural PE teachers in China:

1. Professional Competence reflects the teacher's self-assessment of their ability to fulfill core subject-specific duties under rural conditions. This encompasses not only general pedagogical skills but also the perceived capability to design and deliver effective PE curricula with limited facilities and equipment, to manage large classes with varying fitness baselines commonly found in rural schools, and to implement safety protocols in often non-standard sporting environments.
2. Professional Satisfaction denotes the affective and evaluative dimension derived from one's work. For rural PE teachers, this satisfaction is closely tied to finding purpose and value despite systemic challenges. It includes the sense of accomplishment from improving student health outcomes in underserved communities, the recognition (or struggle for recognition) of PE's importance within a resource-strapped school, and the resilience to maintain commitment in the face of the subject's frequent marginalization in both urban and rural educational priorities.
3. Personal Competence refers to the teacher's perception of their broader personal attributes that enable professional functioning. In the rural context, this dimension gains specific relevance: it involves self-efficacy in building rapport with students who may be left-behind children, skills in engaging with rural communities to promote physical activity, and the personal resilience required to cope with professional isolation and limited peer support.

Factor correlations were as follows:

Professional Competence (Factor 1) and Personal Competence (Factor 3): *r* = 0.71.

Professional Competence (Factor 1) and Professional Satisfaction (Factor 2): *r* = 0.60.

Professional Satisfaction (Factor 2) and Personal Competence (Factor 3): *r* = 0.59.

#### 2.3.2 Work engagement

Work engagement was measured using a 16-item, three-dimensional scale adapted by Li et al. (2015) from the Utrecht Work Engagement Scale (Schaufeli et al., 2002). The dimensions included: Vigor (6 items), e.g., “When I get up in the morning, I feel like going to school.”

Dedication (5 items), e.g., “I am enthusiastic about my job.”

Absorption (5 items), e.g., “When I am working, I forget everything else around me.”

All items were rated on a 5-point Likert scale (1 = strongly disagree, 5 = strongly agree). In this study, Cronbach's alpha for the subscales ranged from 0.82 to 0.88. Moreover, the CFA results showed that the three-dimensional model had acceptable to excellent goodness-of-fit indices (*x*^2^ = 261.15, *x*^2^/*df* = 2.24, CFI = 0.96, TLI = 0.95, RMSEA = 0.046, SRMR = 0.050).

#### 2.3.3 Perception of TPD

Given the distinctive nature of TPD activities, which encompass various forms of training, learning, and practical exercises, the perceptions of these components were collectively measured as perception of TPD. This study specifically evaluated professional training from both subjective and objective dimensions. The objective dimension, focusing on the frequency of professional training, was assessed through a single survey item. For the subjective dimension, which measured the perceived quality of professional training, this study adapted measurement items from the works of Seşen and Ertan (2022), employing the following five survey questions: (1) The training content covers the specific skills, work standards, procedures, objectives, and responsibilities required for my job position; (2) The skills acquired through professional training can meet my job requirements; (3) The training helps me apply the acquired skills to my work; (4) Professional training contributes to improving my work performance; (5) The training has played a positive role in my teaching career.

A confirmatory factor analysis (CFA) was conducted to verify the unidimensional structure of this 5-item scale. The model fit indices were acceptable: χ^2^/*df* = 2.89, CFI = 0.98, TLI = 0.97, RMSEA = 0.051, SRMR = 0.019. All factor loadings were significant and exceeded 0.70. The scale demonstrated excellent internal consistency in this study, with a Cronbach's alpha of 0.93.

### 2.4 Statistical analyses

All analyses were performed using SPSS 23.0 for descriptive statistics and Pearson correlations, and AMOS 23.0 for SEM with maximum likelihood estimation. Model fit was evaluated using χ^2^/*df* ratio (< 3 acceptable, < 2 excellent), CFI/TLI (>0.90 acceptable, >0.95 excellent), RMSEA (< 0.08 acceptable, < 0.06 excellent), and SRMR (< 0.10 acceptable, < 0.08 excellent; Kline, 2001). Mediation effects were tested using 5,000 bootstrap samples to generate 95% confidence intervals (significant if excluding zero). For multi-group SEM comparisons, we examined four levels of measurement invariance (configural, metric, scalar, and strict) using ΔCFI < 0.01 and ΔRMSEA < 0.015 criteria (Cheung and Rensvold, 2002). Partial measurement invariance was applied when full invariance wasn't achieved. MLR robust estimation addressed non-normal data, with TYPE = COMPLEX correcting for cluster effects. Path significance was determined via bias-corrected bootstrap 95% CIs (2,000 resamples), with Benjamini–Hochberg FDR correction (*p* < 0.05) for multiple comparisons. Total effects and mediation proportions ([indirect/total] × 100%) were calculated. Model comparisons prioritized ΔCFI over Δχ^2^ to avoid large-sample sensitivity.

## 3 Results

### 3.1 Common method bias test

Since this study employed a cross-sectional survey design, design, potential common method bias due to common rater and measurement context was considered (Podsakoff et al., 2003). We implemented both procedural and statistical remedies to assess and control for its potential influence. First, procedural controls were applied during data collection. Second, two statistical tests were conducted.

1. Harman's Single-Factor Test: An exploratory factor analysis including all items from the key constructs (perception of TPD, professional self-concept, and work engagement) was performed. The unrotated solution showed that the first factor accounted for 38.7% of the total variance, below the critical threshold of 40%, suggesting CMB was not a predominant concern.

2. Unmeasured Latent Method Factor (ULMF) Technique: Following Podsakoff et al. (2003), we employed a more rigorous confirmatory approach. We first tested the original measurement model (Model A), which yielded the following fit indices: χ^2^/*df* = 2.24, RMSEA = 0.053, SRMR = 0.023, CFI = 0.899, TLI = 0.816. Subsequently, a method factor accounting for the common variance across all items was added to construct Model B. The fit indices for Model B were: RMSEA = 0.087, SRMR = 0.041, CFI = 0.910, TLI = 0.891.

The comparison of the two models revealed acceptable differences: ΔRMSEA = 0.034 (< 0.05), ΔSRMR = 0.018 (< 0.05), ΔCFI = 0.011 (< 0.10), ΔTLI = 0.075 (< 0.10). The small magnitude of these differences, coupled with the low variance explained by the method factor, indicates that common method bias did not significantly distort the measurement model or the substantive relationships under investigation.

### 3.2 Descriptive statistics and group differences

The means (M), standard deviations (SD), and correlation coefficients of the three focal variables are presented in the main table (Table 2), with all key variables demonstrating moderate intercorrelations (*r* = 0.42–0.58). One-way ANOVA with *post hoc* Scheffé tests revealed significant differences (*p* < 0.05, η^2^ = 0.12–0.29) across teaching experience, pedagogical background, and training frequency groups on all study variables, justifying the subsequent multi-group analyses.

**Table 2:** Descriptive statistics and correlation results.

**Variables**	***M***	***SD***	**1**	**2**	**3**
Perception of TPD	24.28	4.573			
Professional self-concept	97.39	11.771	0.420^**^		
Work engagement	64.45	4.522	0.483^**^	0.579^**^	

^**^p < 0.01.

### 3.3 Test of hypotheses

SEM demonstrated acceptable model fit (CFI = 0.899, TLI = 0.985, SRMR = 0.023, RMSEA = 0.053), with all hypothesized paths showing statistically significant standardized regression coefficients: perception of TPD → work engagement (β = 0.176, *p* < 0.01), perception of TPD → professional self-concept (β = 0.644, *p* < 0.001), and professional self-concept → work engagement (β = 0.472, *p* < 0.001), thereby supporting hypotheses H1–H3.

### 3.4 Mediation analysis

The bootstrap analysis with 5,000 resamples (95% bias-corrected confidence intervals) revealed a significant indirect effect of perception of TPD on work engagement through professional self-concept [indirect effect = 0.304, 95% CI (0.174, 0.457)]. The total effect was 0.480 (direct effect = 0.176 + indirect effect = 0.304), with the mediation proportion reaching 63.3%, confirming professional self-concept's significant partial mediating role (H4 supported).

### 3.5 Multi-group SEM analysis

#### 3.5.1 Measurement invariance testing

To examine the measurement equivalence of the TPD quality and professional identity model across subgroups (gender, disciplinary background, teaching experience, and training frequency), multi-group structural equation modeling (MG-SEM) was conducted (Marsh et al., 2006). Following a sequential constraint strategy: Configural invariance (identical factor structure across groups), Metric invariance (equal factor loadings), Scalar invariance (equal indicator intercepts).

Model comparisons were based on fit index differences (Δχ^2^, ΔCFI, AIC/ECVI; Little, 2013). For disciplinary background, training frequency and teaching experience groups, the metric invariance model showed acceptable fit [Δχ^2^ (Δ*df* ) = 19.982(12), *p* = 0.067, ΔCFI = 0.003] with minimized AIC/ECVI values, supporting equal factor loadings across groups. At least metric invariance was established, indicating no systematic bias in latent variable relationships.

#### 3.5.2 Moderating effects on the mediation model

MG-SEM revealed significant path differences in “perception of TPD → professional self-concept → work engagement” across teacher subgroups (Table 3).

**Table 3:** Multi-group analysis results.

**Variables**	**Group**	**PTPD → WE**	**PTPD → PS**	**PS → WE**
Pedagogical background	Education majors	0.182^***^	0.675^***^	0.439^***^
	Non-education majors	0.185^***^	0.529^***^	0.467^***^
Teaching experience	< 1 year	−0.129	0.580^***^	0.736^**^
	1–5 year	1.587^**^	0.911^***^	−1.210^*^
	6–15 year	0.109	0.130	0.234
	>15 year	0.207^*^	0.675^***^	0.490^***^
Frequency	1 time/year	0.174^*^	0.635^***^	0.515^***^
	2 times/year	0.157^*^	0.754^***^	0.391^***^
	≥3 times/year	−0.013	0.710^***^	0.493

^***^p < 0.001, ^**^p < 0.01, ^*^p < 0.05, PTPD, perception of TPD; PS, professional self-concept; WE, work engagement.

Education majors demonstrated stronger direct effects (β = 0.675 vs. 0.529 for non-education majors) and more pronounced mediation through professional self-concept (β = 0.439 vs. 0.567).

#### 3.5.3 Teaching experience

One to five years group showed anomalous patterns: strong direct effect (β = 1.587) but negative mediation (β = −1.210), yielding negative total indirect effect (−1.103).

Ten years group exhibited optimal training effects with consistent positive paths (β = 0.207/0.675/0.490) and total effect = 0.538.

Less than one year and 6–10 years groups showed non-significant paths.

#### 3.5.4 Training frequency

One to two training sessions groups showed significant mediation (total effects = 0.502/0.452).

Greater than or equal to three sessions group displayed non-significant direct paths (β = −0.013) and weakened mediation (β = 0.493), suggesting diminishing returns beyond moderate frequency.

#### 3.5.5 Comparative analysis across career stages

The multi-group SEM results reveal distinct, stage-specific patterns in how TPD perceptions translate into work engagement (summarized in Table 3). A comparative analysis highlights a non-linear developmental trajectory:

Novice Teachers (< 1 year): TPD shows no significant immediate impact on work engagement, suggesting a period of absorption and adaptation where formal training is overshadowed by on-the-job socialization.

Early-Career Teachers (1–5 years): TPD exerts strong direct benefits but concurrently triggers a negative mediation effect through professional self-concept. This indicates a phase of high receptivity coupled with professional identity vulnerability and role conflict.

Mid-Career Teachers (6–15 years): The mediation paths are non-significant, indicating a disconnect between standard TPD offerings and the advanced needs of this cohort, leading to a potential “efficacy gap.”

Veteran Teachers (>15 years): TPD demonstrates consistent positive effects through both direct and mediated pathways, suggesting this group can effectively integrate training to reinforce their established professional identity.

## 4 Discussion

This study examines the interplay between TPD perceptions, professional self-concept, and work engagement within a specific context: rural schools in northeastern China, where PE teachers (predominantly male, at 80.1%) commonly navigate challenges such as larger class sizes, limited sports facilities, and a higher prevalence of left-behind children. Against this backdrop, our systematic examination of the mediating mechanism of teachers' professional self-concept in the relationship between perception of TPD and work engagement through SEM. The results demonstrated a total effect of 0.480 for “perception of TPD → work engagement,” with a direct effect of 0.176 and an indirect effect mediated by professional self-concept of 0.304 [95% CI (0.174, 0.457)], accounting for 63.3% of the total effect, thus supporting H4. These findings confirm that the current rural physical education teacher training system effectively facilitates knowledge internalization (β = 0.644) and teaching competency transformation (β = 0.472), ultimately enhancing work engagement by strengthening professional self-efficacy. The results align with Bakker's research (Bakker and de Vries, 2021), indicating that sufficient job resources reduce burnout and improve engagement, with TPD functioning through a “knowledge acquisition-competency development-identity reinforcement” chain mechanism, providing empirical support for rural PE teachers' professional development.

Consistent with existing literature (Heck et al., 2006; Lakshmanan et al., 2011), TPD shows positive correlations with educational preparedness and practical growth, yet our multi-group comparison reveals this relationship is critically moderated by career stage. Teaching experience analysis revealed significant moderating effects with stage-specific characteristics. Among novice teachers (< 1 year), the path “TPD quality → work engagement” was non-significant (β = −0.129, *p* = 0.506), suggesting limited short-term training effects during this critical transition period where pre-service education and imitation of mentors dominate their development. As evidenced by New York City data (Boyd et al., 2008) this finding implies that targeted support (e.g., mentoring programs) for early-career teachers may yield the highest marginal returns on professional development investments. For 1–5 year teachers, while the direct effect was significantly enhanced (β = 1.587), the negative indirect effect (β = −1.210) reflected a complex, stage-specific dynamic. This pattern suggests that for teachers at this early-career stage, high-quality TPD may simultaneously produce two opposing psychological processes. On one hand, it directly boosts work engagement by providing urgently needed skills and resources (the strong direct effect). On the other hand, it may inadvertently trigger a temporary crisis of professional self-concept, leading to the negative mediation. Drawing on career stage theory, teachers in their 1–5 years are transitioning from survival to consolidation, actively forming their professional identity amidst real-world challenges (Rice, 2013). In resource-constrained rural schools, this cohort often faces intense role conflict: they are expected to apply new, idealistic methods from TPD while managing the daily realities of large classes, limited facilities, and complex student needs (e.g., left-behind children). When training content highlights advanced competencies or pedagogical ideals that starkly contrast with their constrained classroom reality, it may not affirm but rather threaten their nascent sense of professional competence. This tension can manifest as the observed negative mediation, where TPD participation temporarily heightens awareness of the gap between aspiration and reality, thereby straining professional self-concept. Furthermore, the pressure to prioritize immediate classroom management and student feedback over long-term professional integration of training can exacerbate this conflict, making it difficult for a positive professional identity to consolidate as a stable mediator. This interpretation aligns with the notion that the development of a robust professional self-concept is non-linear and can involve phases of destabilization before integration.

In contrast, 6–15 year teachers showed non-significant paths (β = 0.109–0.234, *p* > 0.05), this indicates that current TPD programs may be failing this key cohort. We posit two interconnected reasons: first, the training content is likely perceived as too basic, failing to meet their advanced developmental needs; second, there is a profound lack of professional incentive for this group. Having moved beyond foundational skills, these “mid-career” teachers often face a “professional plateau” where routine tasks dominate and growth stagnates. Without advanced challenges—such as opportunities for curriculum innovation, leading peer workshops, or engaging in action research—TPD fails to renew their sense of efficacy or purpose, leading to disengagement. This cohort consequently demonstrates the weakest training effectiveness and highest attrition risk, a finding that underscores a critical gap in the professional development continuum for rural PE teachers, and resonates with observations about variable instructional efficiency across experience levels (Harris et al., 2025).

Training frequency analysis showed an inverted U-shaped relationship, with optimal effects at 1–2 sessions (total effects = 0.502/0.452) but diminishing returns at ≥3 sessions (β = −0.013), attributable to logistical burdens, content repetitiveness, and limited practical relevance as noted by Chen et al. (2023). Therefore, this pattern should be interpreted not as a call for simply reducing training sessions, but as a strong argument for investing in higher-quality, more relevant, and logistically sustainable TPD experiences that teachers perceive as worth their time investment.

Based on these stage-specific findings, a hierarchical TPD system must be implemented. For early-career teachers (< 1 year), the focus should shift from generic training to structured mentorship and practical classroom management support, aiming to mitigate reality shock and facilitate professional socialization. For those in the 1–5 year phase, programs need to explicitly address the identified role conflict by integrating psychological support with skill development, thus helping them navigate the idealism-reality gap and build a resilient professional identity (Rice, 2013). To re-engage the disaffected 6–15 year cohort, TPD must offer advanced, specialized content (e.g., applied sports science, adaptive curriculum design) and formal leadership opportunities (e.g., peer mentoring roles), with clear linkages to career advancement recognition. Finally, veteran teachers (>15 years) should be leveraged as TPD co-designers and instructional leaders, a role that can renew their professional commitment while simultaneously providing the expert guidance lacking for their mid-career colleagues.

These evidence-based recommendations provide concrete pathways for aligning teacher professional development with national strategic goals for rural education, such as those outlined in China's “Rural Revitalization Strategy” and the ongoing efforts to strengthen the teaching workforce in under-resourced areas. To translate these findings into policy, we suggest a shift from one-size-fits-all training mandates toward a more nuanced policy framework that: (1) supports the decentralization of TPD design to provincial or county levels, allowing for adaptation to local resource contexts and specific teacher needs identified in this study; (2) invests in integrated digital-physical TPD delivery platforms to improve accessibility and reduce logistical burdens for teachers in remote schools; and (3) establishes a “TPD credit and incentive system” that formally links the completion of high-quality, stage-specific training to career advancement, salary increments, and professional recognition, thereby directly addressing the critical incentive gaps revealed in our analysis.

## 5 Conclusion

Three key conclusions emerge: (1) Professional self-concept plays a critical mediating role, validating the “knowledge-competency-identity” mechanism; (2) Training effectiveness exhibits stage-specific patterns, peaking for 1–5 year teachers but weakest for 6–15 year cohorts; (3) Training frequency follows an inverted *U*-curve, emphasizing quality over quantity. Practical recommendations include: (1) Differentiated training designs tailored to career stages; (2) Enhanced environmental and institutional support; (3) Content optimization for relevance and progression; (4) Formal integration of training outcomes into career pathways. These insights advance both theoretical understanding and practical strategies for teacher development in rural contexts.

## 6 Limitations and future research

This study has limitations. First, the cross-sectional design precludes causal claims. Although our model is theoretically grounded, reverse causality (e.g., more engaged teachers seeking better TPD) or unmeasured variables (e.g., intrinsic motivation) could explain the observed relationships. Future longitudinal research is needed to establish temporal precedence.

Second, generalizability is constrained by the sample's geographic focus (three northeastern provinces) and gender imbalance (80.1% male), limiting insights into other regions and female teachers. Furthermore, the study's capacity to precisely define the “rural” context is limited by the lack of collected objective school-level data (e.g., student enrollment, sports facility inventory, teacher-student ratio). While we characterize the setting broadly, this omission prevents a nuanced analysis of how specific resource disparities moderate the observed relationships.

Future studies should pursue nationwide sampling and systematically incorporate objective school-level metrics (e.g., specific resource inventories, class sizes). These data would enable a more nuanced analysis of how institutional contexts moderate the stage-specific pathways identified in this study. Building on our findings, priority directions include: (1) designing contextually adaptive TPD programs, and (2) investigating the interplay between institutional constraints and psychosocial drivers of professional self-concept. This will deepen theoretical insight and inform targeted strategies for teacher retention in rural China.

## Data Availability

The raw data supporting the conclusions of this article will be made available by the authors, without undue reservation.
